# Aging-associated DNA methylation changes in middle-aged individuals: the Young Finns study

**DOI:** 10.1186/s12864-016-2421-z

**Published:** 2016-02-09

**Authors:** L. Kananen, S. Marttila, T. Nevalainen, J. Jylhävä, N. Mononen, M. Kähönen, O. T. Raitakari, T. Lehtimäki, M. Hurme

**Affiliations:** Department of Microbiology and Immunology, School of Medicine, University of Tampere, Tampere, Finland; Gerontology Research Center, Tampere, Finland; Fimlab Laboratories, Tampere, Finland; Departments of Clinical Physiology, Tampere University Hospital and University of Tampere School of Medicine, Tampere, Finland; Research Centre of Applied and Preventive Cardiovascular Medicine and the Department of Clinical Physiology and Nuclear Medicine, University of Turku and Turku University Hospital, Turku, Finland; Department of Clinical Chemistry, University of Tampere School of Medicine, Tampere, Finland

**Keywords:** Aging-associated, DNA methylation, EWAS, CpG sites, Adulthood, Hypermethylation, Blood cell type heterogeneity

## Abstract

**Background:**

Chronological aging-associated changes in the human DNA methylome have been studied by multiple epigenome-wide association studies (EWASs). Certain CpG sites have been identified as aging-associated in multiple studies, and the majority of the sites identified in various studies show common features regarding location and direction of the methylation change. However, as a whole, the sets of aging-associated CpGs identified in different studies, even with similar tissues and age ranges, show only limited overlap. In this study, we further explore and characterize CpG sites that show close relationship between their DNA methylation level and chronological age during adulthood and which bear the relationship regardless of blood cell type heterogeneity.

**Results:**

In this study, with a multivariable regression model adjusted for cell type heterogeneity, we identified 1202 aging-associated CpG sites (a-CpGs, FDR < 5 %), in whole blood in a population with an especially narrow age range (40 - 49 years). Repeatedly reported a-CpGs located in genes *ELOVL2*, *FHL2*, *PENK* and *KLF14* were also identified. Regions with aging-associated hypermethylation were enriched regarding several gene ontology (GO) terms (especially in the cluster of developmental processes), whereas hypomethylated sites showed no enrichment. The genes with higher numbers of a-CpG hits were more often hypermethylated with advancing age. The comparison analysis revealed that of the 1202 a-CpGs identified in the present study, 987 were identified as differentially methylated also between nonagenarians and young adults in a previous study (The Vitality 90+ study), and importantly, the directions of changes were identical in the previous and in the present study.

**Conclusions:**

Here we report that aging-associated DNA methylation features can be identified in a middle-aged population with an age range of only 9 years. A great majority of these sites have been previously reported as aging-associated in a population aged 19 to 90 years. Aging is associated with different types of changes in DNA methylation, clock-like as well as random. We speculate that the a-CpGs identified here in a population with a narrow age-range represent clock-like changes, as they showed concordant methylation behavior in population spanning whole adulthood as well.

**Electronic supplementary material:**

The online version of this article (doi:10.1186/s12864-016-2421-z) contains supplementary material, which is available to authorized users.

## Background

The epigenome includes DNA methylation (DNAmet), post-translational histone modifications and chromatin remodeling. Tens of millions of nucleotides referred to as CpG sites, which are prone to DNAmet, exist in the haploid human genome. Furthermore, the genome-wide DNAmet profile is maintained through cell divisions. DNA methyltransferases apply methyl groups on CpG sites to form 5-methylcytosine, whereas demethylation may occur either passively due to dysfunction of the transferring enzyme or actively through 5-hydromethylcytosine formation. Genomic regions spanning approximately 0.5 kilobases with a high density of CpG sites are called CpG islands, and these are commonly localized near transcription start sites. CpG sites in such islands are often less methylated; thus, the genes are available for initiation of transcription. Moreover, DNAmet plays crucial roles in gene expression by not only blocking the promoter region but also altering the activities of regulatory elements, such as enhancers and insulators. Alternatively, gene body methylation may influence alternative splicing [1, 2]. Thus, the cell identity is in part determined and maintained by a cell type-specific genome-wide methylation pattern, which may therefore be used in the laboratory as a marker to characterize the cell types [3–5].

The genome-wide DNAmet profile of the cell changes; DNAmet patterns are altered in diseases, such as Alzheimer disease, cancer and type 2 diabetes, and are also influenced by the accumulating effects of environmental factors such as toxin exposure and diet [1, 6, 7]. Single CpG sites undergo hypo- and hypermethylation either randomly by stochastic factors or via more systematic mechanisms [1]. For example, exposure to environmental factors such as smoking induces hypomethylation of a well-characterized single CpG site in the gene *F2RL3;* this represents an example of a non-random change in DNAmet because the magnitude of the change is dose and exposure-time dependent [8, 9].

Furthermore, the epigenome is modified by the biological aging process. As also Heyn et al. [10] reported and Zampieri et al. [1] reviewed, in general, aging induces a decrease in average DNA methylation level genome-wide (global hypomethylation). This was demonstrated by whole-genome bisulfite sequencing of newborns and centenarians with as high as ~90 % genomic coverage. The comparison of methylation states between the two extremes of the human lifespan also revealed how the systematic methylation patterns of the CpG sites are eventually lost and how inter-individual differences increase with advanced age. In addition, hypermethylation in regions near promoters can cause down-regulation of essential genes that influence vitally important pathways; Heyn et al. [10] reported that aging-accelerated hypermethylation events occurred in 13 % of the CpG sites among the millions of sites in the genome. Therefore, methylation alterations may be considered as one important factor in the development of aging-associated diseases [1, 10].

Many studies have addressed the aging-associated DNAmet changes in blood cells using Illumina array technology-based methods, which cover 27000 or 485000 CpG sites in the genome [1]. The methylation levels of specific CpG sites are known to be associated with chronological aging in a wide variety of tissues [11–13]. However, as a whole, the sets of aging-associated CpGs identified in different studies, even with comparable tissues and age ranges, show limited overlap. Only few EWASs on age have taken the cell type heterogeneity into account [14–17]. We and others [4] hypothesize that lack of cell type adjustment may have potentially distorted the results obtained, and this may have contributed to the lack of concordance observed between the studies.

In this study, we aimed to discover and characterize regions where the DNAmet levels are associated with chronological age (a-CpGs) in a middle-aged population (aged 40–49 years) through analysis where the cell type heterogeneity was adjusted for. Middle-aged individuals were selected from the Young Finns Study (YFS) [18] follow-up in 2011; the selection in the present study is a balanced sample (i.e. the number of subjects in each age group was equal and the groups had similar sex-distribution), and it therefore provides an excellent opportunity to inspect the effects of aging on DNA methylome. Furthermore, this sample comprises individuals in an extremely narrow age range of only nine years. The subjects’ DNA methylomes were characterized using Illumina Infinium HumanMethylation450 BeadChips and the cell type heterogeneity and sex were adjusted for in the analysis.

Additionally, our findings were interpreted together with compatible data obtained using the same 450BeadChip technology, including our previous results obtained from an EWAS on age (The Vitality 90+ Study, V90+), in which the subjects’ ages ranged from 19 to 90 years [15], as well as other results compiled by Steegenga et al. [19]. The results from the YFS were interpreted by considering that rates of aging-associated DNAmet changes fluctuate, especially during the growth period before adulthood and at the end of the lifespan [11, 20]. Accordingly, the a-CpGs found in the YFS that overlap with those established from adult samples with wider age ranges, such as V90+ study, may be speculated to be DNAmet regions with constant rate of change throughout adulthood. Thus, we aimed to explore the a-CpGs where level of methylation changes in a clocklike fashion throughout adulthood from those that show a more random aging-associated pattern.

## Results

### Aging-associated alterations in DNA methylation

In this study, the genome-wide DNAmet levels in whole blood samples of middle-aged individuals were measured using 450BeadChip technology. The sample heterogeneity (i.e., the proportions of CD8T and CD4T cells, monocytes, granulocytes, and NK and B cells) were estimated by comparing DNAmet profiles to the reference dataset [4] (Additional file 1: Figure S1). The cell type proportions were verified as important determinants of variation in DNAmet using Spearman’s correlation analysis, in which the cell type proportions were correlated with the main principal components (PCs). The PCs were defined with principal component analysis (PCA) from the DNAmet data without cell subtype adjustment (Additional file 2: Table S1a). The analysis revealed that PC1 to PC6 together explained a large proportion (24 %) of the variance in the DNA methylome data. Among those PCs, several PCs had considerable large (-0.5 > *r* >0.5) correlation coefficients; thus, adjustments for the cell type proportion in the analysis were mandatory. The hypothesis whether DNAmet level of a CpG site is associated with chronological age was tested at each CpG site using generalized linear regression analysis (‘beta regression’), where sex and cell type proportions were adjusted for.

We found 1202 a-CpGs (i.e. CpG sites where age was a statistically significant variable in the multivariable regression model, FDR < 5 %) in middle-aged individuals (aged 40–49 years), of which 622 (52 %) were hypomethylated and 580 (48 %) were hypermethylated with advancing age. These hypo- and hypermethylated sites were annotated on 440 and 437 genes, respectively. Lists of the most significant aging-associations in YFS are shown in Tables 1 and 2 and in Additional file 3: Table S4. Frequently reported CpG sites (summarized by Steegenga et al. [19]) located in the *ELOVL2* (cg16867657, cg24724428 and cg21572722), three sites in the *FHL2* (cg06639320, cg22454769 and cg24079702), two sites in the *PENK* (cg16219603, cg16419235), and two sites in the *KLF14* (cg08097417, cg09499629 and cg07955995) were also identified as hypermethylated in the present study.

**Table 1:** The top 20 hypermethylated a-CpGs in middle-aged individuals. The hypermethylated and hypomethylated a-CpGs are shown separately in Tables 1 and 2, respectively. The top-ranking hypermethylated a-CpGs were selected with the following criteria: 1) direction of the association based on the value of beta regression (denoted as ‘betareg’) estimate of age; 2) more than one hit identified per gene (q-value < 0.05 which corresponds to false discovery rate <5 %) and 3) the top-ranking *p*-values. The full list of a-CpGs is shown in Additional file 3: Table S4. The q-value denotes the Benjamini-Hochberg-corrected *p*-value

ProbeID	Gene name	CHR	Coordinate	Betareg estimate of age	q-value
cg16867657	*ELOVL2*	6	11152863	0.022	0.00E + 00
cg24724428	*ELOVL2*	6	11152874	0.021	4.80E-07
cg21572722	*ELOVL2*	6	11152880	0.013	3.46E-06
cg06639320	*FHL2*	2	105382171	0.018	3.46E-06
cg00059225	*GLRA1*	5	151284550	0.013	5.13E-06
cg08097417	*KLF14*	7	130069673	0.020	1.87E-05
cg22454769	*FHL2*	2	105382199	0.021	5.03E-05
cg07553761	*TRIM59*	3	161650671	0.016	6.12E-05
cg01588592	*ETV3L*	1	155335949	0.011	1.14E-04
cg11176990	*LOC375196*	2	39041037	0.014	1.54E-03
cg09499629	*KLF14*	7	130069676	0.018	1.54E-03
cg22158769	*LOC375196*	2	39041043	0.020	2.43E-03
cg18898125	*NEFM*	8	24826286	0.012	2.49E-03
cg21911021	*ZIK1*	19	62786823	0.020	3.07E-03
cg27217742	*RGS12*	4	3335078	0.013	3.07E-03
cg17737681	*DLX1*	2	172660382	0.015	3.29E-03
cg24079702	*FHL2*	2	105382203	0.015	5.99E-03
cg16219603	*PENK*	8	57523140	0.013	7.00E-03
cg23930856	*TFAP2B*	6	50919683	0.013	7.22E-03
cg11152943	*TRAPPC9*	8	141318170	0.013	7.57E-03

**Table 2:** The top 20 hypomethylated a-CpGs in middle-aged individuals. The hypermethylated and hypomethylated a-CpGs are shown separately in Tables 1 and 2, respectively. The top-ranking hypomethylated a-CpGs were selected with the following criteria: 1) direction of the association based on the value of beta regression (denoted as ‘betareg’) estimate of age; 2) more than one hit identified per gene (q-value < 0.05 which corresponds to false discovery rate < 5 %) and 3) the top-ranking *p*-values. The full list of a-CpGs is shown in Additional file 3: Table S4. The q-value denotes the Benjamini-Hochberg-corrected *p*-value

ProbeID	Gene name	CHR	Coordinate	Betareg estimate of age	q-value
cg00791074	*MTHFD1L*	6	151227862	-0.018	7.51E-04
cg18618815	*COL1A1*	17	45630323	-0.018	5.99E-03
cg14169886	*PRDM16*	1	3101709	-0.014	5.99E-03
cg01820374	*LAG3*	12	6752344	-0.014	9.24E-03
cg19421125	*LAG3*	12	6753117	-0.022	1.02E-02
cg14829066	*NTRK3*	15	86360145	-0.013	1.49E-02
cg03290281	*C6orf195*	6	2577606	-0.021	1.49E-02
cg05561193	*DCLK2*	4	151218492	-0.017	1.96E-02
cg20249566	*NWD1*	19	16691739	-0.024	1.97E-02
cg23928726	*PEX10*	1	2334858	-0.014	1.97E-02
cg20007894	*SCAND3*	6	28648421	-0.019	2.08E-02
cg16355231	*PEX10*	1	2334839	-0.019	2.14E-02
cg15058210	*HDAC4*	2	239861814	-0.018	2.16E-02
cg06030846	*TMEM108*	3	134581182	-0.011	2.16E-02
cg25994988	*UBASH3B*	11	122157592	-0.011	2.16E-02
cg18345924	*NCAM2*	21	21294102	-0.016	2.18E-02
cg00638021	*COL1A1*	17	45622061	-0.013	2.26E-02
cg19344626	*NWD1*	19	16691749	-0.024	2.36E-02
cg01288258	*ITFG2*	12	2792128	-0.011	2.41E-02
cg05221385	*TAF10*	11	6590080	-0.010	2.43E-02

Interestingly, similar to correlation analysis results shown in Additional file 2: Table S1a, the cell type proportions were important determinants of variation in DNAmet levels of the 1202 a-CpGs as well (Additional file 2: Table S1b). In this second correlation analysis, the PCs were defined with PCA from DNA methylation data of the 1202 a-CpGs (aging-associated CpG sites, FDR < 5 %); methylation data in PCA were not adjusted for the cell subtype heterogeneity. Correlation analysis revealed that PC1-PC6 determined more than 50 % of variance in methylation levels of these a-CpGs and these PCs correlated clearly with age and the cell counts. It is also worth of mentioning that of the 1202 a-CpGs in our initial aging-association analysis, there were 526 multivariable regression models (corresponding 526 CpG sites) where all cell count variables (monocytes, granulocytes, NK, CD8T and CD4T cells) were detected as statistically significant (FDR < 5 %) predictors of DNA methylation levels.

The importance of the cell count considerations was explored with an additional set of regression models, where the DNA methylation level in each CpG site genome-wide was explained with age and sex only while the cell counts were not adjusted for. In this analysis, only 56 sites were classified as aging-associated (FDR < 5 %) and these sites were all included to the original pool of 1202 a-CpGs. The 56 a-CpGs are pointed out in the Additional file 3: Table S4.

**Fig. 1:**
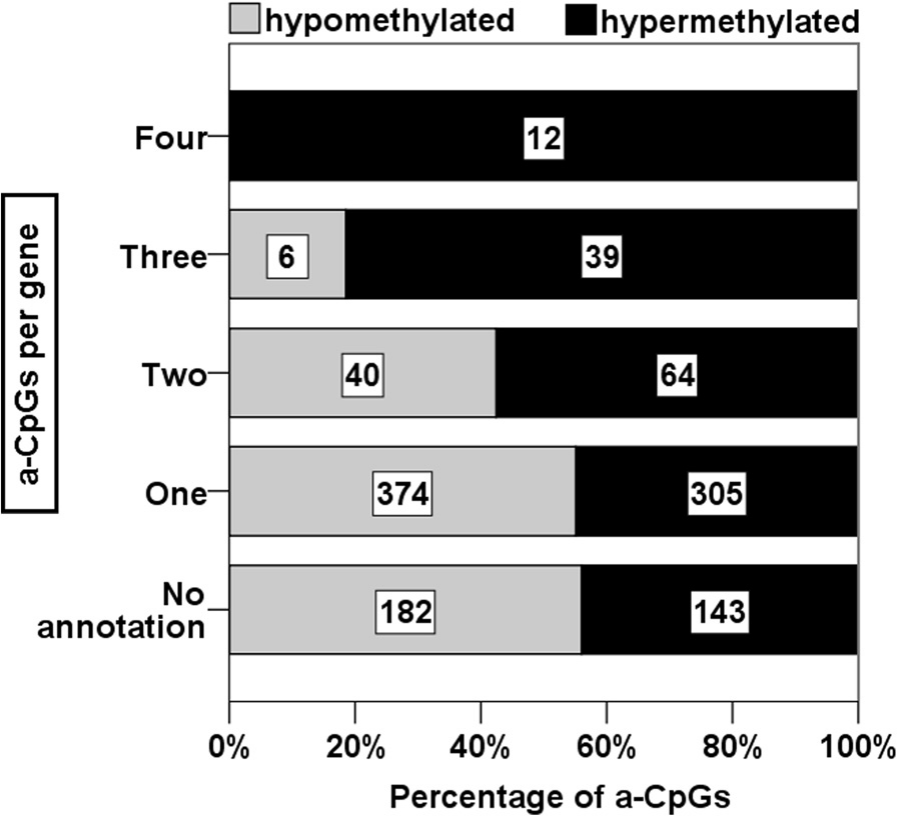
Numbers of aging-associated CpG sites (hits) per gene in regard to hypermethylation and hypomethylation is visualized as bars. Aging-associated hypermethylation was more frequent within genes with more association hits. First, the genes were categorized into groups based on the number of hypermethylated or hypomethylated a-CpG hits per gene. Next, the frequencies of hypermethylated and hypomethylated a-CpGs within the groups were calculated. The number of a-CpGs for each group is shown inside each bar

Aging-associated hypermethylation and hypomethylation differ in their features. The exploration of aging-associations in the YFS revealed that hypermethylation was more frequent within genes with more association hits as shown in Additional file 4: Table S5 and Fig. 1). Specifically, there were 70 genes in total either with more than one hypomethylated or more than one hypermethylated a-CpGs per gene. Of those, 22 genes comprised more than one hypomethylated a-CpGs per gene and 48 genes comprised more than one hypermethylated a-CpGs per gene as shown in Additional file 4: Table S5.

Next, the genomic locations of the a-CpGs were investigated, revealing that 388 of the 1202 a-CpGs were located on CpG islands rather than island shores, shelves or non-island regions, and a majority (N = 331) of those were hypermethylated (Additional file 1: Figure S2). The remaining sites were distributed to shores, shelves and non-island regions with opposite manner as shown in Additional file 1: Figure S2; the aging-associated hypomethylation was more abundant on those regions. The a-CpG locations on genes were also investigated; no enrichment of a-CpGs was detected in the regions of 3′ untranslated regions (UTRs), 5′UTRs or close distances to transcription start sites or gene bodies (Additional file 1: Figure S3a and b). The distributions of the a-CpGs on chromosomes were also investigated; hypermethylated a-CpGs were over-represented on chromosome 18, whereas hypomethylated sites were not enriched on any chromosome (hypergeometric test, nominal *p*-value of 0.05) (Additional file 1: Figure S3c). In addition, we ensured using visual examination that there were no spatial local cluster(s) of a-CpGs on Chr-18.

### Sex specificity of the aging-associated CpG sites

To evaluate the sex specificity of the aging-associations, an interaction model with variables corresponding to sex, age and the interaction of sex and age (age*sex) was constructed. No sex-specific a-CpGs were identified, as analysis revealed that no interaction term had a false discovery rate (FDR) below 5 % (q-value < 0.05) in the interaction models. Furthermore, we analyzed women (N = 111) and men (N = 73) separately as well: sex-specific a-CpGs were explored among all CpG sites with an multivariable regression model (‘beta regression’) where age and cell type proportion variables were used to predict DNA methylation level in each CpG site. These analyses revealed that there were 105 and 173 a-CpGs (FDR < 5 %) among men and women, respectively; these CpG sites were all included to our original pool of 1202 a-CpGs which were detected using whole sample (N = 184). Importantly, as shown in Additional file 1: Figure S5, when the directions of change among the 1202 a-CpGs were cross-compared between men and women (without *p*-value cut-off), all sites, except one, showed concordant behavior regarding hypermethylation or hypomethylation during aging (i.e. whether the estimate of age variable in the regression model was negative or positive value). This behavior was also identical to the directions of change among the 1202 a-CpGs in the initial analysis (N = 184). As a conclusion, these results were in line with our interaction analysis: there were no significantly sex-specific a-CpGs among middle-aged individuals.

### Functional roles of a-CpGs in the YFS

The gene ontology (GO) functions and processes of the genes with a-CpGs were investigated using the Gene Ontology enRIchment anaLysis and visuaLizAtion (GOrilla) tool [21]. The analysis was conducted separately for genes with hypermethylated a-CpGs and for hypomethylated a-CpGs (N = 440 and N = 437, respectively). The analysis revealed an unambiguous differences between hypo- and hypermethylated a-CpGs, as 73 GO process terms and to 8 GO function terms were enriched to genes with hypermethylated a-CpGs (Tables 3 and 4, respectively; Additional file 2: Table S2.), whereas there was no enrichment of terms among the genes with hypomethylated a-CpGs (Bonferroni-adjusted *p*-value threshold of 0.05). The most statistically significant processes were anatomical structure development (GO:0048856, *p* = 1.02*10^-11^) and morphogenesis (GO:0009653, *p* = 5.02*10^-10^), both of which cluster under the term ‘developmental process’.

**Table 3:** Several GO process terms were enriched within genes with hypermethylated a-CpGs in the analysis with GOrilla [21, 43]. This table represents the main clusters of processes (53 redundant GO terms were filtered out of 73 terms using REViGO [44]). The full list of processes is shown in Additional file 2: Table S2

GO term	Description of the process	*p*-value (-log10)
GO:0048856	Anatomical structure development	10.9914
GO:0050794	Regulation of cellular process	8.9788
GO:0007389	Pattern specification process	8.2343
GO:0032502	Developmental process	8.2041
GO:0009893	Positive regulation of metabolic process	8.0511
GO:0044708	Single-organism behavior	7.5544
GO:0035108	Limb morphogenesis	7.5544
GO:0003002	Regionalization	7.3585
GO:0051239	Regulation of multicellular organismal process	7.301
GO:0006357	Regulation of transcription from RNA polymerase II promoter	7.2248
GO:0065007	Biological regulation	7.1675
GO:0007610	Behavior	7.08
GO:0048598	Embryonic morphogenesis	7.0778
GO:0048518	Positive regulation of biological process	6.8761
GO:0048519	Negative regulation of biological process	6.7122
GO:0008285	Negative regulation of cell proliferation	6.4921
GO:0048523	Negative regulation of cellular process	5.8827
GO:0010842	Retina layer formation	5.8041
GO:0051961	Negative regulation of nervous system development	5.7423
GO:0032774	RNA biosynthetic process	5.4225

**Table 4:** GO function terms were enriched within genes with hypermethylated a-CpGs in the analysis with GOrilla. Table contains the full list of enriched GO function terms (Bonferroni-adjusted *p* < 0.05) obtained from analysis with GOrilla [21, 43]

GO term	Description of the function	*p*-value (-log10)
GO:0043565	Sequence-specific DNA binding	10.001
GO:0000981	Sequence-specific DNA binding RNA polymerase II transcription factor activity	7.322
GO:0001071	Nucleic acid binding transcription factor activity	6.721
GO:0003700	Sequence-specific DNA binding transcription factor activity	6.721
GO:0003677	DNA binding	6.625
GO:0005326	Neurotransmitter transporter activity	5.148
GO:0005488	Binding	4.967

In addition, Pscan [22] was used to predict whether there were common regulators for groups of genes. The hypermethylation-associated genes were predicted to be regulated by 11 common transcription factors (Additional file 2: Table S3), several of which were zinc coordinating. For hypomethylation-associated genes, no common transcription factors were found. A large proportion of the 11 regulators of genes with hypermethylated a-CpGs in the YFS were zinc coordinating, and four (E2F1, EGR1, SP1, TFAP2A) were identical to those identified in the V90+ study [15].

### Comparisons to other studies

**Fig. 2:**
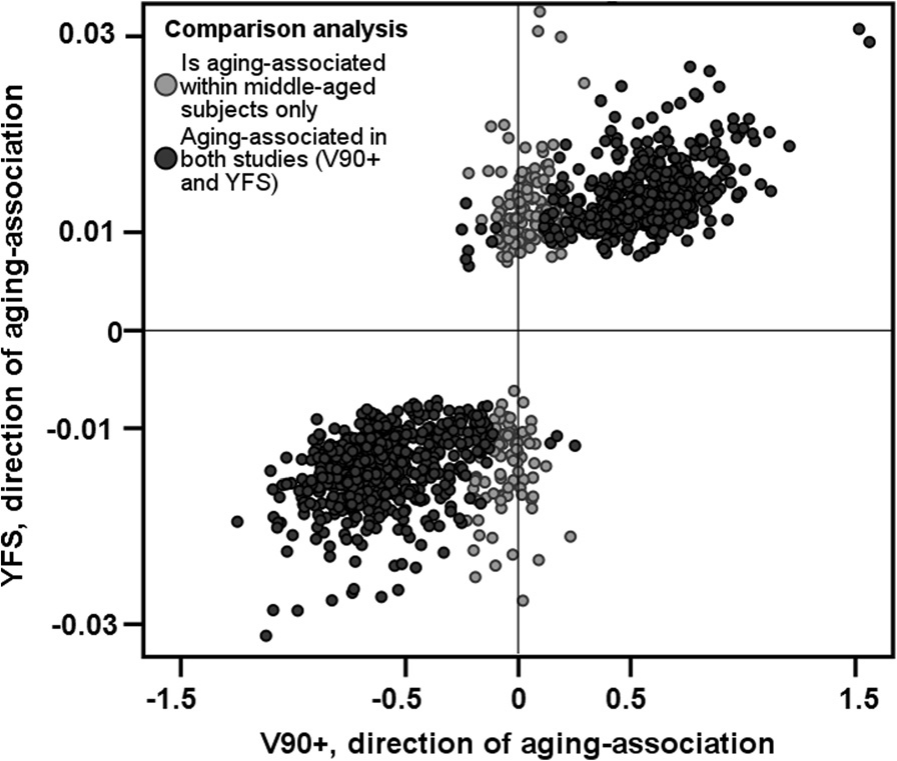
The direction of aging-association in 1202 a-CpGs is visualized as scatterplot. Each dot corresponds to single a-CpG; directions of associations correspond to estimates of age which are fetched from the regression models. Of 1202 sites, 987 CpG sites were similarly associated with aging in both the YFS and in the V90+ study. The analyses in both studies were adjusted for leukocyte cell subtype proportions, and the studies consisted of the samples with distinct age ranges: the YFS comprised 40 to 49 years old subjects whereas the V90+ study consisted of 19–30-year-old individuals and nonagenarians. The corresponding data illustrated in the Fig. 2 is presented in Additional file 3: Table S4

In the explorative cross-comparison analysis, the a-CpGs identified in middle-aged individuals of the YFS were compared to aging-associated DNA methylome alterations between nonagenarians and 19–30-year-old individuals evidenced in our previous study (the V90+ study) [15]. The a-CpGs identified in the V90+ study were strongly associated with aging while the cell type heterogeneity was adjusted for in the analysis. A total of the 1202 a-CpGs established in the YFS cohort, 999 a-CpGs were also aging-associated in the V90+ sample (FDR < 5 %, Additional file 3: Table S4). Of these 999 a-CpGs, 464 (46 %) were hypermethylated, and 535 (54 %) were hypomethylated with advancing age. Furthermore, in 987 of the overlapping 999 a-CpGs the direction of the aging-associated change was the same: in the present and in the V90+ study, 455 a-CpGs were hypermethylated, and 532 were hypomethylated with advancing age (Fig. 2).

**Fig. 3:**
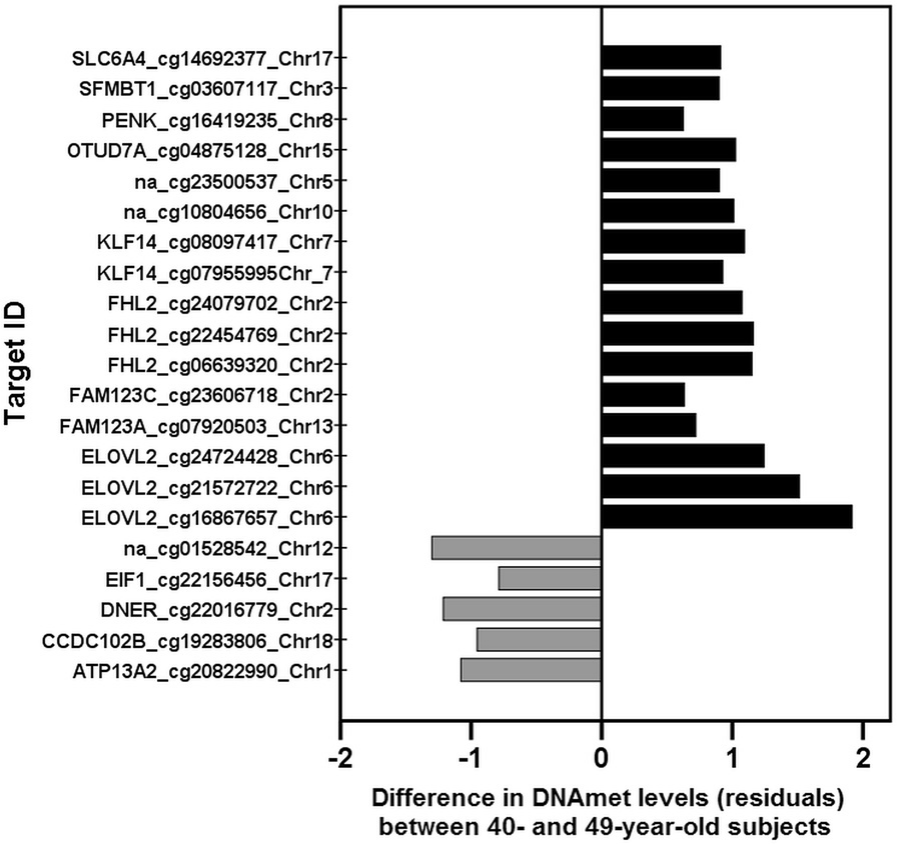
The top 21 most commonly reported a-CpGs and their direction of association with aging. The top 21 a-CpGs were selected with following criteria: the a-CpG was identified in present study and in the V90+ study, as well as in two or more other studies (Hannum et al. [13], Garagnani et al. [12] or Florath et al. [23]); the sites were reported as aging-associated in blood samples and the data were obtained using 450 BeadChip technology. Methylation level differences in YFS between the highest and the lowest age groups (between 40- and 49-year-old individuals; calculated from the medians of residuals after adjusting for effects of sex and cell type proportions), are illustrated as bars. The bars are colored according to the hypomethylation or hypermethylation status (grey = hypomethylated, black = hypermethylated). Gene annotation is shown for each bar, where applicable (na = no gene annotation). The corresponding data is presented in Additional file 3: Table S4

Finally, a-CpGs that were characterized from whole blood samples as aging-associated using 450 BeadChip technology and previously reported by Hannum et al. (number of hits, 89) [13], Garagnani et al. (number of hits, 9) [12] and Florath et al. (number of hits, 162) [23] and presented as summary table in Steegenga et al [19] were further compared with our data. The corresponding age of the samples ranged between 19 -101, 9–83 and 50–75 years, respectively. The comparison revealed 21 common CpG sites out of the 999 a-CpGs in two or more studies in addition to the YFS and the V90+ study (Fig. 3).

## Discussion

In this study, we identified 1202 a-CpGs where the DNAmet level was associated with aging in middle-aged individuals (i.e. with an age range of 40 to 49 years), in whom the growth and development of youth has ended yet old age and its associated diseases had not begun. Of the 1202 a-CpGs, 622 (52 %) ​were hypomethylated, and 580 (48 %) were hypermethylated with advancing age, with annotations on 440 and 437 different genes, respectively. In general, the functional features of these aging-associated sites are mostly similar to those identified from cohorts with larger age differences. Our study highlights also that a large number of sites undergo aging-associated DNAmet level changes throughout adulthood and we speculate that a great proportion of those probably change with a clock-like manner.

A large fraction of the DNAmet sites are altered during the lifespan, as shown by previous studies performed using 450BeadChip technology [15, 24] and whole-genome bisulfite sequencing [10]. Furthermore, the rates of these changes may fluctuate at different stages of the lifespan. Studies have shown that a-CpGs behave differently during the growth period before adulthood and at the end of the lifespan [11, 20]. Nonetheless, there are genes (*ELOVL2*, *SFMBT1*, *KLF14*, *PENK*, and *FHL2*) with CpG sites that are consistently detected as being aging-associated despite of differences in sample tissue types or age distributions [11–13, 15]; notably, these genes were also identified in the present study as being aging-associated (Tables 1 and 2; Additional file 3: Table S4). However, a recent meta-analysis on three DNAmet data sets obtained using 450BeadChip illustrated discrepancies in the lists of regions where DNAmet levels were altered during the entire human lifespan, ranging from 0 to 100 years of age [2]. Because blood sample heterogeneity has been shown to have a great impact on EWASs [4, 15], our speculation is that the discrepancies might be due to the presence of different cell types.

In the primary analysis, we aimed to identify a-CpGs in middle-aged individuals representing general population with age range of only one decade. Then, we cross-compared the results to those obtained with similar analysis pipeline from a population aged 19 to 90 years (Vitality 90+ study) [15]. Among the 1202 a-CpGs characterized from the YFS with an age range of nine years, 987 sites had an identical association direction as detected in the Vitality 90+ study, as shown in Fig. 2 and in Additional file 3: Table S4. We hypothesize that sites displaying aging-associated methylation changes in both populations possibly represent sites where the change in DNA methylation follows a clock-like pattern. We further speculate that the non-overlapping CpG sites identified in the population with a wider age range (19 to 90 years of age) may possibly represent sites where the aging-associated change is accelerated in either early or late adulthood; the a-CpGs identified only when comparing group of nonagenarians to young adults may represent changes that reflect e.g. aging-associated pathologies or accumulation of aging-associated impairments.

As aging influences the immune system of men and women differently and as the risk rates of several diseases between sexes are unequal [25, 26], 1) an interaction analysis was performed to address the sex specificity of a-CpGs, and 2) the aging-associations were also evaluated in separate analyses among men and women. These analyses revealed no sex-specific single a-CpGs; thus, the identified a-CpGs are universally altered in both men and women. These results are in accordance with our previous results from the V90+ study, in which the DNAmet states of nonagenarians were compared with 19–30-year-old individuals [15], and with results published by others [24, 27]. However, studies have shown that as a whole, the DNA methylomes of males age more rapidly than those of females [13, 28].

Aging-accelerated hypomethylation may be thought as an erosion-like event, whereas hypermethylation may be thought as an actively guided process. In practice, the difference between these features is manifested, for example, through the enrichment of GO terms for groups of genes and for signaling pathways [1, 15]. The distinct roles of the methylation status were demonstrated in the present study with the numbers of a-CpG hits in a gene, as we observed notable enrichment of hypermethylation events located in genes with more than one a-CpG (Fig. 1). The functional roles of genes with a-CpGs were established by GO term enrichment analysis, which revealed obvious difference between hypo- and hypermethylated a-CpGs, even though the analysis was conducted with an equal number of genes in the GO term analyses. A high number of GO terms were enriched to genes with hypermethylated a-CpGs (Tables 3 and 4; Additional file 2: Table S2), whereas there was no GO term enrichment within genes with hypomethylated a-CpGs. The most statistically significant processes enriched to genes with hypermethylated a-CpGs were ‘anatomical structure development’ and ‘morphogenesis’, both of which cluster under the term ‘developmental process’. The enrichment of hypermethylated a-CpGs to these processes has been reported previously [14, 23, 24, 29]. Reynolds [30] and Yuan [16] reported also that the CpG sites hypermethylated during aging are enriched to common processes and exhibit shared features, whereas hypomethylated a-CpGs are a less homogenous group. Furthermore, age-associated hypermethylation interactome hotspots have been reported [31].

In addition to the details mentioned above, we observed other similar hypermethylation characteristics in the YFS, as those reported in previous studies [1, 15]. For example, the majority (85 % out of 388) of a-CpGs localized in CpG-islands (instead of shores, shelves or other regions) were hypermethylated, and an excess of hypermethylated a-CpGs were also found on chromosome 18. However, there was no enrichment of a-CpGs on chromosome 19. In the V90+ study, the hypermethylated a-CpGs located in the genes encoding zinc-associated proteins were more abundant on chromosome 19 [15], where zinc-finger genes are clustered. The zinc-finger genes (such as *ZNF154*) located in chromosome 19 are proposed to be repressors of endogenous retroviruses (ERVs) [32], and the repressor activity may be disturbed by hypermethylation. Interestingly, CpG sites located in the gene *ZNF154* and almost all other genes encoding zinc-fingers on chromosome 19 were absent from our pool of 1202 a-CpGs. Thus, as the hypermethylation of CpG sites located in genes encoding zinc-fingers was observed in the oldest age group, we hypothesize that rates of methylation level changes at the CpG sites located in ERV repressor genes (e.g. *ZNF154*) may fluctuate throughout the lifespan and that the rates may be enhanced in association with other senescence-related factors. Therefore, it is possible that DNAmet-based dysfunction of the repression system might explain the increased expression of ERVs in old age [33]. Future studies are required to address these questions.

To further inspect the roles of the genes with aging-accelerated DNAmet changes, analysis of the common regulators (transcription factors) of groups of genes with hypermethylated and hypomethylated a-CpGs was conducted with Pscan [22]. The results were again surprisingly concordant with those in the V90+ study. There were 11 regulators with unique identifiers for hypermethylated a-CpGs (Additional file 2: Table S3), whereas hypomethylated a-CpGs had no common regulators. A great proportion of the 11 regulators of genes with hypermethylated a-CpGs in the YFS were zinc coordinating, and four (E2F1, EGR1, SP1, and TFAP2A) were identical to those identified in the V90+ study results [15]. Overall, the results from analysis of the functional roles of the genes with a-CpGs were surprisingly well in line with the observations from the V90+ study and supported the proposition that aging-associated hypermethylation is a more tightly regulated process, whereas aging-associated hypomethylation is induced more by environmental effects and stochastic factors.

Finally, we demonstrated the lack of concordance in previously reported pools of a-CpGs by comparing three published lists of overlapping a-CpGs produced using 450BeadChips from whole blood samples from subjects with age ranges of 50–75, 19–101 and 9–83 [12, 13, 23]. Although 987 of the a-CpGs in the YFS showed similar association directions as in the V90+ study (Fig. 2 and Additional file 3: Table S4), we observed only 61 overlapping a-CpGs in the YFS and the V90+ study, which were also reported as aging-associated in one or more other robustly compatible studies (same sample type and array technology). Of these, only 21 a-CpGs were observed in two or more of the studies in the comparison (Fig. 3). To the best of our knowledge [4, 15], the main factor that contributes to the DNAmet profiles in blood cells is cell type heterogeneity; thus, we speculate that the lack of cell type adjustments may account for the majority of disparity in the cross-comparisons. The results of aging-association analysis and combined PCA-correlation analysis in this study supports our speculation. Cell type heterogeneity should be taken into account when analyzing samples composed of mixed cell types, but a limited number of such studies have been conducted [4, 14–17].

Notably, our study had an obvious limitation, it would substantially benefit from being a follow-up; therefore, future studies are needed. Nevertheless, the analysis is powered by well-designed sample characteristics because each age group was matched by sex and sample size and because adjustments were made for cell type heterogeneity. Thus, the analysis was sensitive enough to detect DNAmet changes within an age range spanning nine years.

## Conclusions

Here we report that aging-associated DNA methylation changes can be identified in a middle-aged population with a narrow age range of 9 years. Aging-associated DNAmet changes are not uniform, but occur due to different reasons, at different rates and directions in different parts of the genome and are not alike in all cell types. Thus, due to this diverse nature of aging-associated DNA methylation changes, all confounding factors should be accounted for in the analysis, in order to obtain comparable results. Our results support the notion that cell type heterogeneity should be adjusted for when analyzing tissues consisting of mixed cell types. Moreover, our results imply that considerable proportion of DNAmet changes show clock-like behavior throughout adulthood.

## Methods

### Study population

The Young Finns study (YFS) comprises a series of six cohorts, representing general population, born in 1962, 1965, 1968, 1971, 1974 and 1977 from five cities with university hospitals in Finland (Helsinki, Kuopio, Oulu, Tampere and Turku) [18]. A subsample of 184 individuals was randomly assigned from a follow-up in 2011. The sample collection in 2011 is described in more detail elsewhere [34]. The categories of age in the methylation analysis were 40, 43, 46 and 49 years old, with group sizes of 50, 44, 55 and 35, in which 58 %, 68.2, 56.4 and 60 % were women, respectively. All of the participants were of western European descent. The study followed the guidelines of the Declaration of Helsinki and was approved by the Ethical Review Committee of Turku University Hospital. All participants provided informed consent.

### DNA methylome quantification

#### Sample preparations

Leukocyte DNA of the YFS cohort was obtained from EDTA-blood samples using a Wizard® Genomic DNA Purification Kit (Promega Corporation, Madison, WI, USA) according to the manufacturer’s instructions. Genome-wide DNA methylation levels were obtained using Illumina Infinium HumanMethylation450 BeadChips [35–37] in the Core Facility at the Institute of Molecular Medicine Finland (FIMM), University of Helsinki according to the protocol by Illumina.

The methylation data set was preprocessed identically with a previously described analysis pipeline which was used in the DNA methylation analysis of the V90+ study samples [15, 38, 39]. Briefly, methylation signal data was preprocessed as a methylumiset object using R software (*R* > = 2.15.3) with array-specific algorithms implemented in the R package wateRmelon [40] and BMIQ [38]. The resulting β values ranged linearly from 0 (non-methylated, 0 %) to 1 (completely methylated, 100 %). The quality of DNA samples and methylation data was carefully ensured by standard examinations with principal component analysis (PCA) and visualizations with density plots, boxplots and dotplots. Three of the YFS samples were excluded due to atypically low probe intensities compared with control probe intensities.

The YFS sample was lacking leukocyte cell type characterizations; thus, the proportions were determined by the estimation algorithm implemented in the estimateCellCounts function of the minfi Bioconductor package [4] using R software (*R* > = 2.15.3). The algorithm utilizes the selection of 600 control probes that represents specific signatures of CD8T and CD4T cells, monocytes, granulocytes, and NK and B cells (Additional file 1: Figure S1). The reference data used in the estimation is available in the FlowSorted.Blood.450K Bioconductor package [4].

### Quality control of the DNA methylome data

As the cell type proportions contribute to most of the variation in genome-wide DNAmet [4, 15], the significance of the estimated cell counts in the DNAmet data was investigated by PCA, and the main PCs of DNAmet were correlated with the cell counts (Additional file 2: Table S1a). Spearman’s correlation analysis indicated a clear connection between methylation profiles and estimated cell proportions. Thus, the estimated cell counts as well as the genome-wide methylation data was shown to behave as expected.

As part of the quality control step, a well-known CpG site with phenotype association was selected. Smoking is strongly associated with the hypomethylation of cg03636183, located in the gene *F2RL3* [8, 9]; our data from the YFS replicated this finding, as we observed a difference between daily smokers and others (Wilcoxon rank sum-test, *P* = 2.4*10^-6^; Additional file 1: Figure S4). Analysis with multivariable regression model (function lm() in R) revealed that the cell type heterogeneity, age or sex of the samples did not alter the finding of cg0363618.

### Detection of aging-associated methylation regions

Aging-associated CpG sites, the a-CpGs, were explored using a generalized linear regression model, referred to as the ‘variable dispersion beta regression’ in an iterative manner for each methylation locus (CpG site). The age (categories of 40, 43, 46 and 49) was employed as a variable to predict the site-specific methylation outcome in the form of a β value (ranging from 0 to 1); this was done in each equation using the mean model and a linker function of *logit*. The cellular heterogeneity was adjusted in the initial multivariable regression analyses: in addition to age and sex variables, variables corresponding to each estimated blood cell subtype proportion (CD8T and CD4T cells, monocytes, granulocytes, NK and B cells; all ranging linearly from 0 to 1) were included to the regression models as predictors of DNA methylation level. Additionally, sex-specific a-CpGs were explored among all CpG sites using two approaches: 1) with an interaction model where age, sex, sex*age and cell type proportion variables were used to predict DNA methylation level, and 2) with an regression model where age and cell type proportion variables were used to predict DNA methylation level separately for men and women. Furthermore, to explore the relevance of the cell count considerations in the regression analyses, an additional set of age-association analyses was performed. In these regression models, the DNA methylation level of each CpG site was explained with age and gender variables only and the cell proportions were not adjusted for. The analyses were performed using R software (*R* > = 2.15.3), and the regression analyses were mainly conducted with algorithms implemented in the betareg package [41]. The nominal Benjamini-Hochberg adjusted *p*-value (q-value) was set to 0.05. The a-CpGs were annotated based on the assembly provided by the R package, FDb.InfiniumMethylation.hg19 [42]. For the purpose of visualization in Fig. 3, standardized weighted residual values of the methylation levels were extracted for each CpG site from regression models in which only sex and cell type proportion variables were set as predictors.

### Analysis of the functional roles of a-CpGs

The enriched gene ontology (GO) terms of the genes with a-CpGs were discovered using GOrilla [21, 43], and the significant terms were further clustered by REViGO [44]. The GOrilla analysis was performed for the process, function and component categories with two un-ranked lists, of which the first list comprised genes with hypomethylated or hypermethylated a-CpGs (Additional file 3: Table S4), and the second comprised the genes in the background (N = 20,902; analysis date, 9.3.2015). Furthermore, the prediction of common transcription factors of the groups of genes with either hypermethylated or hypomethylated a-CpGs (as two separate analyses) was conducted using Pscan with the default settings (JASPAR database; analysis date, 10.3.2015) [22]. The nominal *p*-value was set to at the Bonferroni-corrected value of 0.05 in each analysis.

### Availability of supporting data

The methylation data presented in this manuscript have been submitted to the Gene Expression Omnibus (GEO) database (http://www.ncbi.nlm.nih.gov/geo/) under the accession number GSE69270.

## Additional files

Additional file 1: Figures S1-S5. 1) A figure of estimated proportions of CD8T, CD4T, NK, B cell, monocyte and granulocyte cells of peripheral blood samples in YFS. Proportions are visualized as boxplots, categorized by age group and organized to separate panels by sex. 2) A figure of aging-associated CpG site locations in regard to CpG islands (CGIs). Number of aging-associated CpG sites are visualized with stacked bars. 3) A figure (a-c) presenting locations of a-CpGs. 4) A figure showing results for association of DNA methylation level in cg03636183 with smoking. 5) A figure presenting sex specificity of the aging-associated CpG sites (a-CpGs). (DOCX 357 kb) Additional file 2: Tables S1-S3. 1) Two summary tables (a and b) of the results from Spearman correlation analyses between age, the cell counts and the first principal components (PCs). PCs were defined from either the whole methylation data or 1202 a-CpGs using PCA. 2) A table of the GO terms of the bio processes that are enriched to genes with aging-associated CpG-sites. 3) A table of common transcription factors for genes with hypermethylated a-CpGs characterized using Pscan. (DOCX 31 kb) Additional file 3: Table S4. A full table of 1202 a-CpGs with detailed information. (XLSX 183 kb) Additional file 4: Table S5. A summary table where the 70 genes with more than one hypomethylated or more than one hypermethylated a-CpGs per gene are presented. (XLSX 15 kb)

## Abbreviations

a-CpGs: aging-associated CpG sites (FDR < 5 %)
DNAmet: DNA methylation
ERV: endogenous retrovirus
EWAS: epigenome-wide association studies
PC: principal component
UTR: untranslated region
V90+: The Vitality 90+ Study
YFS: Young Finns study
