# Supplementary material for: Aging-associated DNA methylation changes in middle-aged individuals: the Young Finns study
Source: BMC Genomics. 2016 Feb 9;17:103. doi: 10.1186/s12864-016-2421-z (PMC4746895; doi:10.1186/s12864-016-2421-z)

**Additional file 1**

**Figure 1. Estimated proportions of CD8T, CD4T, NK, B cell, monocyte and granulocyte cells in YFS pheripheral blood visualized as boxplots which are categorized by age group (40, 43, 49 and 49) and organized to separate panels by gender.** The cell types were determined from the DNA with EstimateCellCounts algorithm using Bioconductor package implemented in minfi.


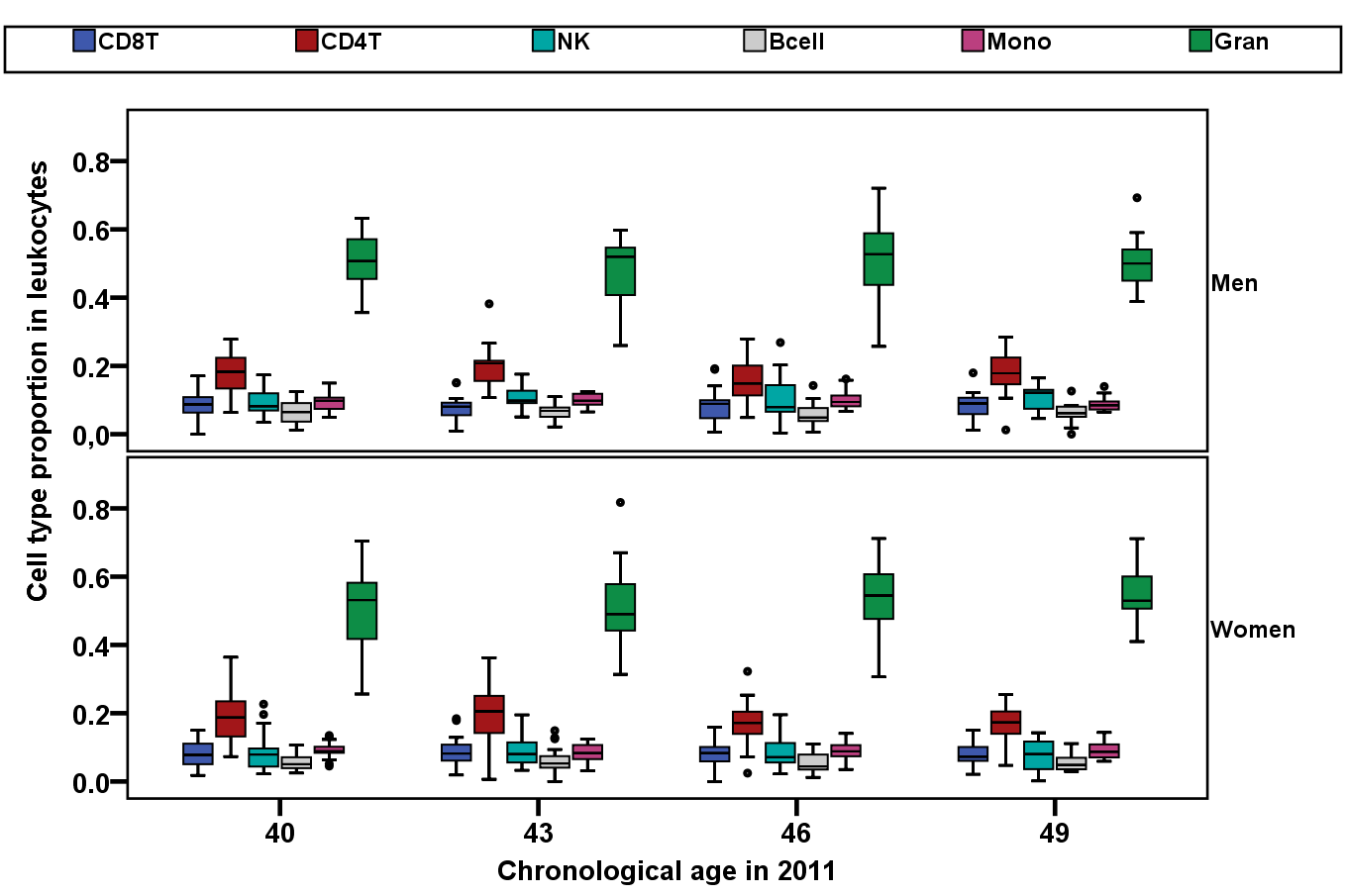


**Figure 2. Aging-associated CpG site location in regard to CpG islands (CGIs).** Number of aging-associated CpG sites are visualized with stacked bars where each location (‘S shore’, ‘S helf’, ‘N shelf’, ‘N shore’, ‘island’ and other regions denoted as ‘non-CGI’) on the Y-axis are categorized by hypermethylation (back color) and hypomethylation (grey color) statuses. The numbers are shown inside each stack bar.


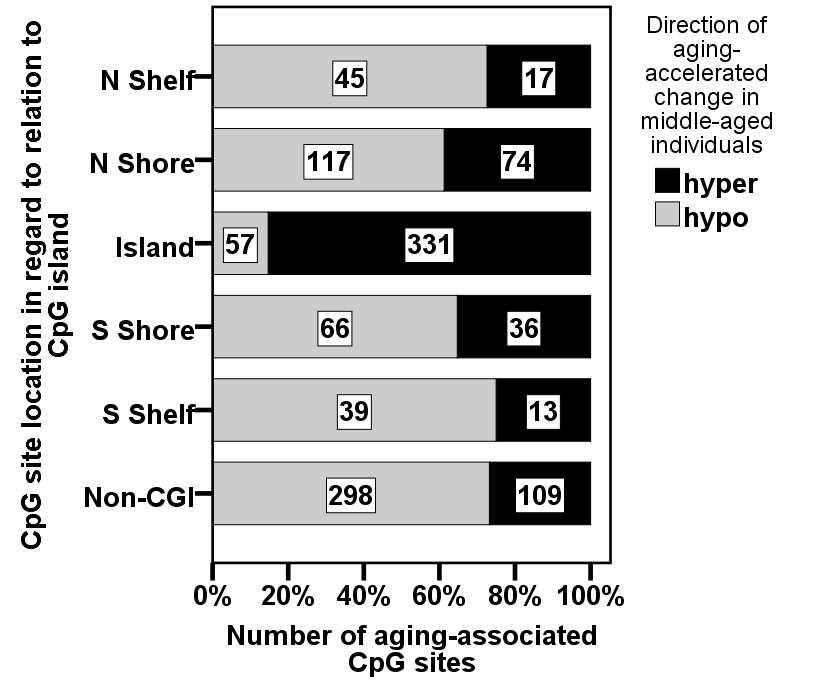


**Figure 3. Locations of a-CpGs.** The proportions of a-CpGs are presented as bars. The locations of hyper- and hypomethylated a-CpGs are shown in regard to A) CpG islands (CGIs), B) genes and C) chromosomes. ‘Array’ denotes the total percentage of probes on the array. Bar colors are explained in the figure legend.


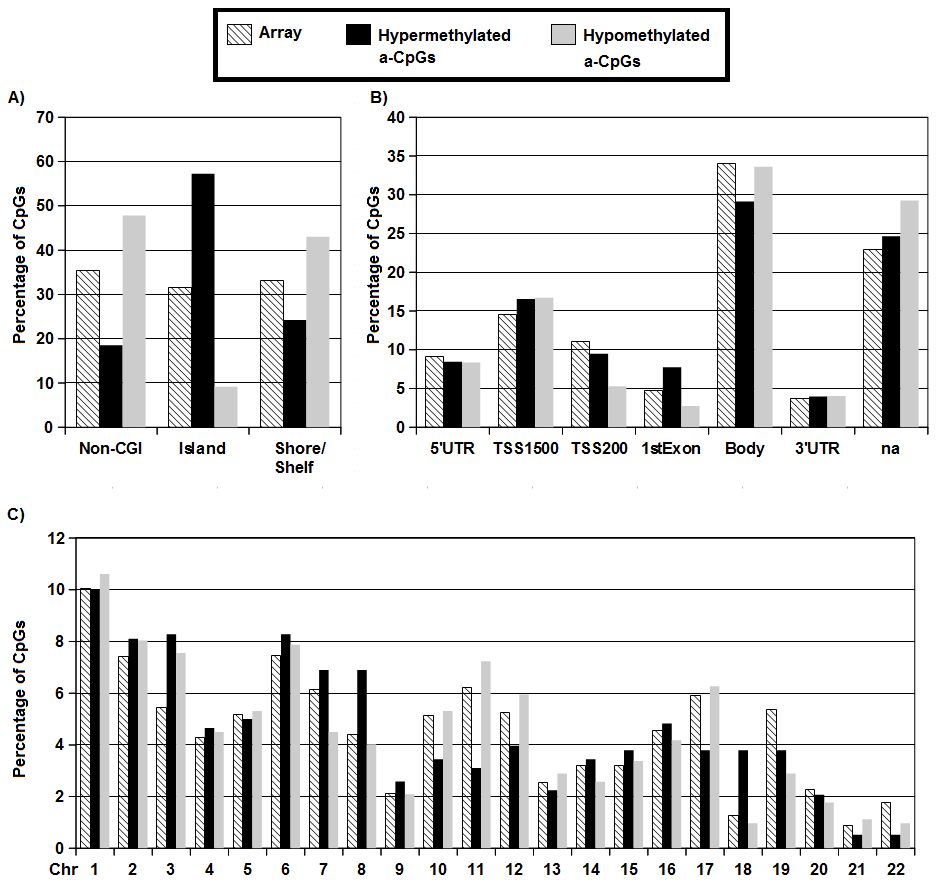


**Figure 4.** **Results for association of DNA methylation level in cg03636183 with smoking.** The association with smoking remained significant even when age, the cell subtype proportions and sex were adjusted for in the regression analysis: smoking was the most significant predictor in the multivariable regression model (β=-0.105, P=1.16x10^-23^). Participants’ smoking status was determined with a question of daily smoking (yes or no): there were 21 daily smokers among the 184 participants.

**
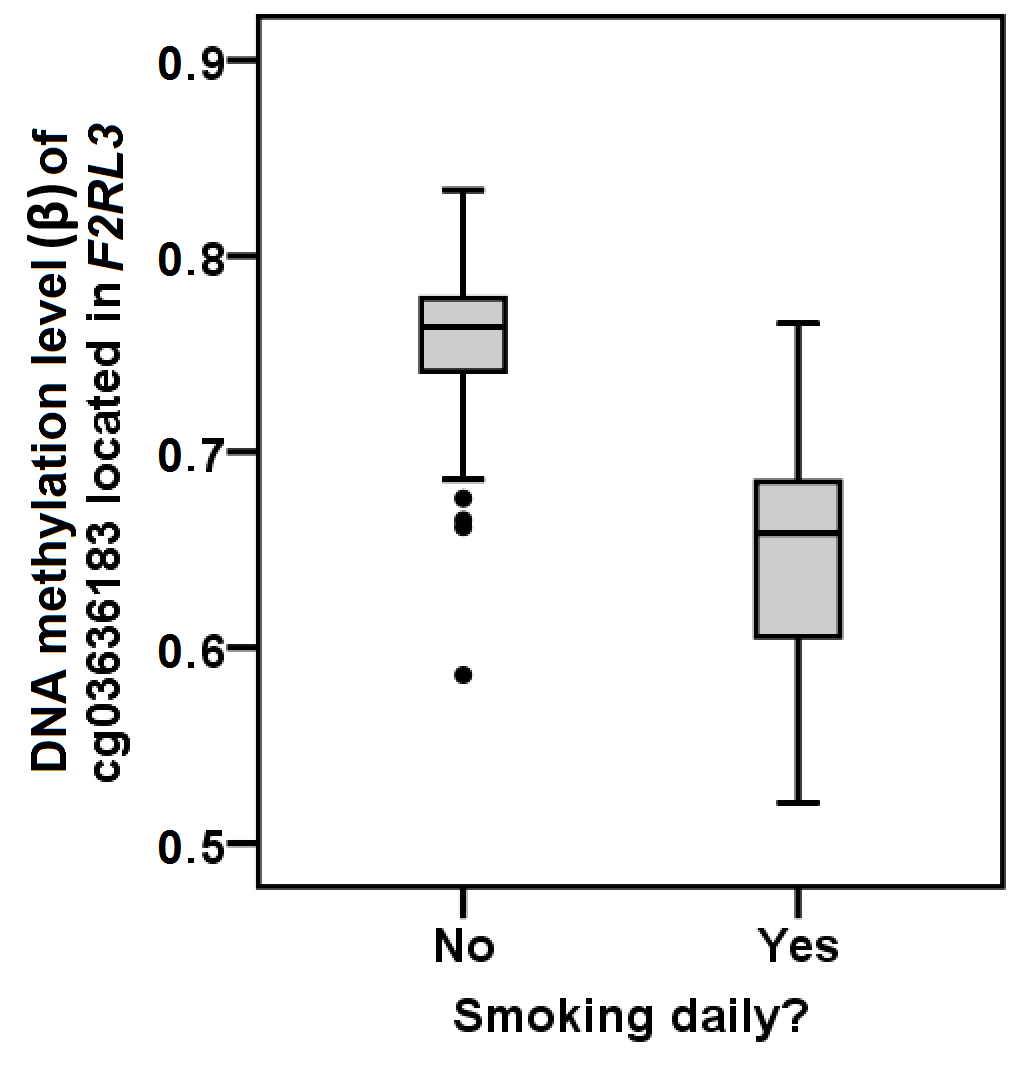
**

**Figure 5. Sex specificity of the aging-associated CpG sites (a-CpGs).** Sex-specific a-CpGs were explored in separate analyses for men (N=73) and women (N=111) among all CpG sites (genome-wide) with a multivariable regression model (‘beta regression’) where age and cell subtype proportion variables were used to predict DNA methylation level in each CpG site. The original pool of 1202 a-CpGs was detected using whole YFS sample (N=184, age range from 40 to 49). The directions of associations (without p-value cut-off) in the original pool of 1202 a-CpGs identified separately among men and women are illustrated in a scatterplot: a-CpGs showed concordant behavior (i.e. whether they lose or gain DNA methylation) during aging for both genders.


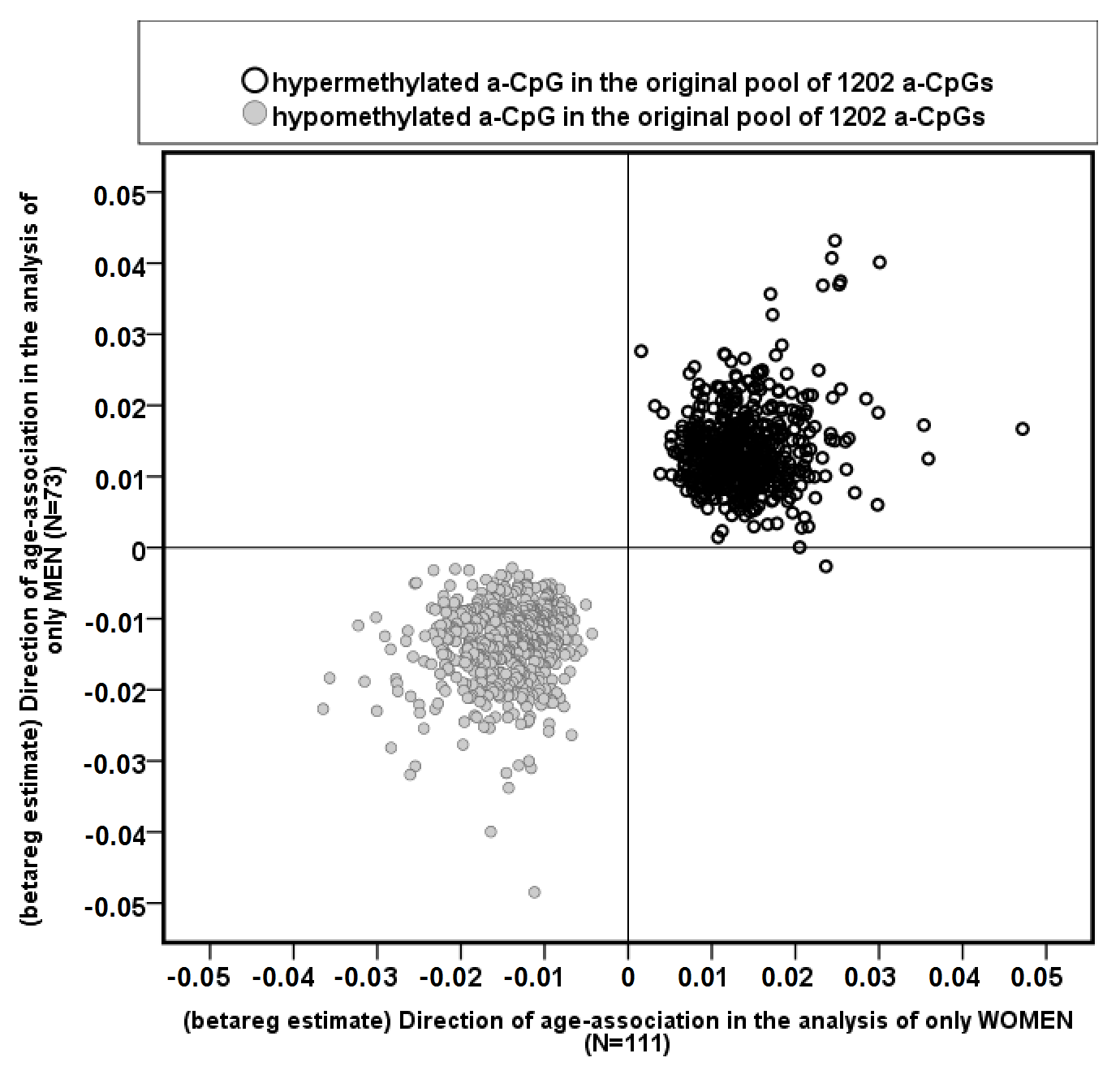

Supplement: Additional file 1: Figures S1-S5. — 1) A figure of estimated proportions of CD8T, CD4T, NK, B cell, monocyte and granulocyte cells of peripheral blood samples in YFS. Proportions are visualized as boxplots, categorized by age group and organized to separate panels by sex. 2) A figure of aging-associated CpG site locations in regard to CpG islands (CGIs). Number of aging-associated CpG sites are visualized with stacked bars. 3) A figure (a-c) presenting locations of a-CpGs. 4) A figure showing results for association of DNA methylation level in cg03636183 with smoking. 5) A figure presenting sex specificity of the aging-associated CpG sites (a-CpGs). (DOCX 357 kb) [file 12864_2016_2421_MOESM1_ESM.docx]
