# Supplementary material for: Aging-associated DNA methylation changes in middle-aged individuals: the Young Finns study
Source: BMC Genomics. 2016 Feb 9;17:103. doi: 10.1186/s12864-016-2421-z (PMC4746895; doi:10.1186/s12864-016-2421-z)
Supplement: Additional file 2: Tables S1-S3. — 1) Two summary tables (a and b) of the results from Spearman correlation analyses between age, the cell counts and the first principal components (PCs). PCs were defined from either the whole methylation data or 1202 a-CpGs using PCA. 2) A table of the GO terms of the bio processes that are enriched to genes with aging-associated CpG-sites. 3) A table of common transcription factors for genes with hypermethylated a-CpGs characterized using Pscan. (DOCX 31 kb) [file 12864_2016_2421_MOESM2_ESM.docx]

**Additional file 2**

**Tables 1a and b. The results from Spearman correlation analyses (N=184) between the cell counts, the six first principal components (PCs) and age in 2011 (40-49 years). In Table 1a:** The PCs were defined with PCA from the whole DNA methylation data (Illumina 450Beadchip coverage). PC1-PC6 determined great proportion (24%) of variance in DNA methylation levels. **In Table 1b:** The PCs were defined with PCA from the DNA methylation data of only 1202 a-CpGs (aging-associated CpG sites, FDR<5%). PC1-PC6 determined more than 50% of variance in methylation levels of these a-CpG. As shown in both tables, in these analyses, there were several PCs with high (-0.5>r>0.5) correlation coefficients. For clarity, ‘r’ denotes for Spearman’s rho and ‘P’ for the statistical significance (2-tailed).

| **Table 1a** |  | **PC1  (9.4%)** | **PC2  (4.8%)** | **PC3 (3.1%)** | **PC4 (2.8%)** | **PC5 (2.4%)** | **PC6 (1.7%)** |
| --- | --- | --- | --- | --- | --- | --- | --- |
| **CD8 T cells** | **r** | -0.684 | 0.109 | 0.175 | 0.047 | -.036 | 0.043 |
|  | **P** | **1.07E-26** | 1.39E-01 | **1.76E-02** | 5.26E-01 | 6.31E-01 | 5.65E-01 |
| **CD4 T cells** | **r** | -0.399 | -0.237 | 0.241 | -0.613 | 0.392 | 0.083 |
|  | **P** | **1.97E-08** | **1.22E-03** | **9.63E-04** | **2.33E-20** | **3.81E-08** | 2.60E-01 |
| **NK cells** | **r** | -0.637 | -0.048 | -0.174 | 0.088 | -0.087 | -0.006 |
|  | **P** | **2.51E-22** | 5.21E-01 | **1.85E-02** | 2.35E-01 | 2.38E-01 | 9.31E-01 |
| **B cells** | **r** | -0.27 | 0.197 | 0.108 | -0.319 | 0.217 | -0.005 |
|  | **P** | **2.06E-04** | **7.32E-03** | 1.45E-01 | **1.00E-05** | **3.08E-03** | 9.43E-01 |
| **Monocytes** | **r** | 0.307 | -0.028 | -0.186 | 0.075 | -0.024 | 0.045 |
|  | **P** | **2.22E-05** | 7.07E-01 | **1.13E-02** | 3.10E-01 | 7.49E-01 | 5.40E-01 |
| **Granylocytes** | **r** | 0.819 | 0.135 | -0.128 | 0.431 | -0.24 | -0.076 |
|  | **P** | **8.77E-46** | **6.74E-02** | **8.43E-02** | **9.76E-10** | **1.01E-03** | 3.08E-01 |
| **Age in 2011** | **r** | -0.036 | 0.071 | -0.027 | 0.174 | -0.041 | -0.096 |
|  | **p** | 6.23E-01 | 3.35E-01 | 7.13E-01 | **1.83E-02** | 5.81E-01 | 1.94E-01 |

| **Table 1b** |  | **a-CpG**  **PC1  (31.5%)** | **a-CpG**  **PC2  (9.5%)** | **a-CpG**  **PC3 (7.2%)** | **a-CpG**  **PC4 (2.5%)** | **a-CpG**  **PC5 (1.8%)** | **a-CpG**  **PC6 (1.4%)** |
| --- | --- | --- | --- | --- | --- | --- | --- |
| **CD8 T cells** | **r** | -0.625 | -0.229 | -0.135 | 0.117 | 0.200 | -0.052 |
|  | **P** | **2.57E-21** | **1.78E-03** | **6.75E-02** | 1.15E-01 | **6.44E-03** | 4.83E-01 |
| **CD4 T cells** | **r** | -0.355 | -0.470 | 0.594 | 0.165 | -0.220 | 0.034 |
|  | **P** | **7.58E-07** | **1.68E-11** | **6.42E-19** | **2.51E-02** | **2.75E-03** | 6.45E-01 |
| **NK cells** | **r** | -0.629 | 0.023 | -0.081 | -0.180 | 0.170 | -0.087 |
|  | **P** | **1.12E-21** | 7.58E-01 | 2.72E-01 | 1**.45E-02** | **2.08E-02** | 2.40E-01 |
| **B cells** | **r** | -0.203 | -0.371 | 0.013 | 0.038 | -0.262 | -0.064 |
|  | **P** | **5.72E-03** | **2.25E-07** | 8.60E-01 | 6.10E-01 | **3.32E-04** | 3.85E-01 |
| **Monocytes** | **r** | 0.285 | 0.132 | -0.005 | -0.135 | -0.064 | -0.049 |
|  | **P** | **8.65E-05** | **7.40E-02** | 9.47E-01 | **6.68E-02** | 3.86E-01 | 5.10E-01 |
| **Granylocytes** | **r** | 0.745 | 0.452 | -0.344 | -0.044 | 0.058 | 0.030 |
|  | **P** | **9.11E-34** | **1.25E-10** | **1.75E-06** | 5.56E-01 | 4.31E-01 | 6.89E-01 |
| **Age in 2011** | **r** | -0.208 | 0.479 | -0.256 | 0.331 | -0.431 | -0.114 |
|  | **p** | **4.60E-03** | **6.22E-12** | **4.61E-04** | **4.41E-06** | **1.05E-09** | 1.23E-01 |

**Table 2.** **The GO terms of the bio processes enriched to genes with ageing-associated CpG-sites.** Table consists of terms that associate with genes with statistical significance of Bonferroni-corrected p-value less than 0.05.

| **GO term** | **Description of the process** | **P-value** | **q-value (FDR)** |
| --- | --- | --- | --- |
| GO:0048856 | anatomical structure development | 1.02E-11 | 1.35E-07 |
| GO:0009653 | anatomical structure morphogenesis | 5.02E-10 | 3.31E-06 |
| GO:0050794 | regulation of cellular process | 1.05E-09 | 4.60E-06 |
| GO:0048869 | cellular developmental process | 2.74E-09 | 9.04E-06 |
| GO:0050789 | regulation of biological process | 5.10E-09 | 1.35E-05 |
| GO:0007389 | pattern specification process | 5.83E-09 | 1.28E-05 |
| GO:0051960 | regulation of nervous system development | 6.18E-09 | 1.17E-05 |
| GO:0032502 | developmental process | 6.25E-09 | 1.03E-05 |
| GO:0019222 | regulation of metabolic process | 7.15E-09 | 1.05E-05 |
| GO:0009893 | positive regulation of metabolic process | 8.89E-09 | 1.17E-05 |
| GO:0009952 | anterior/posterior pattern specification | 1.29E-08 | 1.55E-05 |
| GO:0010628 | positive regulation of gene expression | 1.33E-08 | 1.47E-05 |
| GO:0050767 | regulation of neurogenesis | 1.70E-08 | 1.73E-05 |
| GO:0060284 | regulation of cell development | 1.85E-08 | 1.74E-05 |
| GO:2000026 | regulation of multicellular organismal development | 2.16E-08 | 1.90E-05 |
| GO:0044708 | single-organism behavior | 2.79E-08 | 2.30E-05 |
| GO:0035107 | appendage morphogenesis | 2.79E-08 | 2.16E-05 |
| GO:0035108 | limb morphogenesis | 2.79E-08 | 2.04E-05 |
| GO:0009887 | organ morphogenesis | 2.88E-08 | 2.00E-05 |
| GO:0044767 | single-organism developmental process | 3.43E-08 | 2.27E-05 |
| GO:0003002 | regionalization | 4.38E-08 | 2.75E-05 |
| GO:0010468 | regulation of gene expression | 4.95E-08 | 2.97E-05 |
| GO:0051239 | regulation of multicellular organismal process | 5.00E-08 | 2.87E-05 |
| GO:0006357 | regulation of transcription from RNA polymerase II promoter | 5.96E-08 | 3.28E-05 |
| GO:0065007 | biological regulation | 6.80E-08 | 3.59E-05 |
| GO:0048513 | organ development | 7.26E-08 | 3.68E-05 |
| GO:0007610 | behavior | 8.31E-08 | 4.06E-05 |
| GO:0048598 | embryonic morphogenesis | 8.36E-08 | 3.94E-05 |
| GO:0048518 | positive regulation of biological process | 1.33E-07 | 6.03E-05 |
| GO:0048519 | negative regulation of biological process | 1.94E-07 | 8.51E-05 |
| GO:0045664 | regulation of neuron differentiation | 2.01E-07 | 8.57E-05 |
| GO:0031323 | regulation of cellular metabolic process | 2.17E-07 | 8.95E-05 |
| GO:0014014 | negative regulation of gliogenesis | 2.61E-07 | 1.04E-04 |
| GO:0030326 | embryonic limb morphogenesis | 2.71E-07 | 1.05E-04 |
| GO:0035113 | embryonic appendage morphogenesis | 2.71E-07 | 1.02E-04 |
| GO:2001141 | regulation of RNA biosynthetic process | 2.72E-07 | 9.96E-05 |
| GO:0008285 | negative regulation of cell proliferation | 3.22E-07 | 1.15E-04 |
| GO:0051252 | regulation of RNA metabolic process | 3.41E-07 | 1.18E-04 |
| GO:0009891 | positive regulation of biosynthetic process | 3.68E-07 | 1.25E-04 |
| GO:1903506 | regulation of nucleic acid-templated transcription | 3.82E-07 | 1.26E-04 |
| GO:0031328 | positive regulation of cellular biosynthetic process | 4.69E-07 | 1.51E-04 |
| GO:0006355 | regulation of transcription0. DNA-templated | 4.88E-07 | 1.53E-04 |
| GO:0048522 | positive regulation of cellular process | 4.96E-07 | 1.52E-04 |
| GO:0050768 | negative regulation of neurogenesis | 5.22E-07 | 1.56E-04 |
| GO:0006351 | transcription0. DNA-templated | 6.20E-07 | 1.82E-04 |
| GO:0097659 | nucleic acid-templated transcription | 6.31E-07 | 1.81E-04 |
| GO:0051254 | positive regulation of RNA metabolic process | 6.97E-07 | 1.96E-04 |
| GO:0009888 | tissue development | 7.16E-07 | 1.97E-04 |
| GO:0051171 | regulation of nitrogen compound metabolic process | 8.33E-07 | 2.24E-04 |
| GO:0045595 | regulation of cell differentiation | 8.79E-07 | 2.32E-04 |
| GO:0045165 | cell fate commitment | 9.00E-07 | 2.33E-04 |
| GO:0060255 | regulation of macromolecule metabolic process | 9.43E-07 | 2.39E-04 |
| GO:0035137 | hindlimb morphogenesis | 1.04E-06 | 2.59E-04 |
| GO:0050793 | regulation of developmental process | 1.16E-06 | 2.85E-04 |
| GO:0009889 | regulation of biosynthetic process | 1.24E-06 | 2.97E-04 |
| GO:0019219 | regulation of nucleobase-containing  compound metabolic process | 1.24E-06 | 2.92E-04 |
| GO:0048666 | neuron development | 1.25E-06 | 2.89E-04 |
| GO:0031326 | regulation of cellular biosynthetic process | 1.26E-06 | 2.87E-04 |
| GO:0035136 | forelimb morphogenesis | 1.28E-06 | 2.85E-04 |
| GO:0048523 | negative regulation of cellular process | 1.31E-06 | 2.87E-04 |
| GO:0048562 | embryonic organ morphogenesis | 1.37E-06 | 2.97E-04 |
| GO:0031325 | positive regulation of cellular metabolic process | 1.46E-06 | 3.10E-04 |
| GO:1902680 | positive regulation of RNA biosynthetic process | 1.52E-06 | 3.18E-04 |
| GO:0010842 | retina layer formation | 1.57E-06 | 3.23E-04 |
| GO:0051961 | negative regulation of nervous system development | 1.81E-06 | 3.67E-04 |
| GO:0045893 | positive regulation of transcription. DNA-templated | 1.83E-06 | 3.65E-04 |
| GO:1903508 | positive regulation of nucleic acid-templated transcription | 1.83E-06 | 3.60E-04 |
| GO:0014013 | regulation of gliogenesis | 2.05E-06 | 3.98E-04 |
| GO:0010557 | positive regulation of macromolecule biosynthetic process | 2.39E-06 | 4.58E-04 |
| GO:0030182 | neuron differentiation | 2.66E-06 | 5.01E-04 |
| GO:0051240 | positive regulation of multicellular organismal process | 3.06E-06 | 5.68E-04 |
| GO:0000122 | negative regulation of transcription  from RNA polymerase II promoter | 3.49E-06 | 6.40E-04 |
| GO:0032774 | RNA biosynthetic process | 3.78E-06 | 6.82E-04 |

**Table 3. Common transcription factors for genes with hypermethylated a-CpGs characterized by Pscan.** The p-value threshold was set to 1.12x10^-7^ (Bonferroni corrected p-value of 0.05).

| **TF_NAME** | **MATRIX_ID** | **Z_SCORE** | **P_VALUE** | **TF category** |
| --- | --- | --- | --- | --- |
| **E2F3** | MA0469.1 | 7.30721 | 1.16E-13 | Winged Helix-Turn-Helix |
| **E2F4** | MA0470.1 | 7.04962 | 7.91E-13 | Winged Helix-Turn-Helix |
| **E2F1** | MA0024.2 | 6.91192 | 2.07E-12 | Winged Helix-Turn-Helix |
| **EGR1** | MA0162.2 | 6.85755 | 3.12E-12 | Zinc-coordinating |
| **NRF1** | MA0506.1 | 6.08198 | 5.30E-10 | other |
| **SP1** | MA0079.2 | 6.01552 | 8.18E-10 | Zinc-coordinating |
| **SP2** | MA0516.1 | 5.82861 | 2.58E-09 | Zinc-coordinating |
| **Egr1** | MA0162.1 | 5.7352 | 4.50E-09 | Zinc-coordinating |
| **TFAP2A** | MA0003.1 | 5.63747 | 8.01E-09 | Zipper-Type |
| **ZBTB33** | MA0527.1 | 5.51643 | 1.58E-08 | Zinc-coordinating |
| **SP1** | MA0079.3 | 5.2215 | 8.31E-08 | Zinc-coordinating |
